# Synthesis of a New Chelating Iminophosphorane Derivative (Phosphazene) for U(VI) Recovery

**DOI:** 10.3390/polym14091687

**Published:** 2022-04-21

**Authors:** Bahig M. Atia, Ahmed K. Sakr, Mohamed A. Gado, Hassan S. El-Gendy, Nagwa M. Abdelazeem, Enass M. El-Sheikh, Mohamed Y. Hanfi, M. I. Sayyed, Jamelah S. Al-Otaibi, Mohamed F. Cheira

**Affiliations:** 1 Nuclear Materials Authority, P.O. Box 530, El Maadi, Cairo, Egypt; dr_bahig.atia@yahoo.com (B.M.A.); mag.nma@yahoo.com (M.A.G.); elgendy_nma@yahoo.com (H.S.E.-G.); elsba3y@hotmail.com (E.M.E.-S.); mokhamed.khanfi@urfu.ru (M.Y.H.); 2 National Research Center (NRC), 33 El-Buhoth Street, Dokki, Cairo 12622, Egypt; nagwamorad@yahoo.com; 3 Institute of Physics and Technology, Ural Federal University, St. Mira, 19, 620002 Yekaterinburg, Russia; 4 Department of Physics, Faculty of Science, Isra University, Amman 11622, Jordan; dr.mabualssayed@gmail.com; 5 Department of Nuclear Medicine Research, Institute for Research and Medical Consultations (IRMC), Imam Abdulrahman Bin Faisal University (IAU), P.O. Box 1982, Dammam 31441, Saudi Arabia; 6 Department of Chemistry, College of Science, Princess Nourah bint Abdulrahman University, P.O. Box 84428, Riyadh 11671, Saudi Arabia; jsalotabi@pnu.edu.sa

**Keywords:** Uranium, N–Hydroxy–N–trioctyl–iminophosphorane (HTIP), solvent extraction, G. Gattar granite, leach liquor

## Abstract

A new synthetic chelating N–hydroxy–N–trioctyl iminophosphorane (HTIP) was prepared through the reaction of trioctylphosphine oxide (TOPO) with N–hydroxylamine hydrochloride in the presence of a Lewis acid (AlCl_3_). Specifications for the HTIP chelating ligand were successfully determined using many analytical techniques, ^13^C–NMR, ^1^H–NMR, FTIR, EDX, and GC–MS analyses, which assured a reasonable synthesis of the HTIP ligand. The ability of HTIP to retain U(VI) ions was investigated. The optimum experimental factors, pH value, experimental time, initial U(VI) ion concentration, HTIP dosage, ambient temperature, and eluents, were attained with solvent extraction techniques. The utmost retention capacity of HTIP/CHCl_3_ was 247.5 mg/g; it was achieved at pH = 3.0, 25 °C, with 30 min of shaking and 0.99 × 10^−3^ mol/L. From the stoichiometric calculations, approximately 1.5 hydrogen atoms are released during the extraction at pH 3.0, and 4.0 moles of HTIP ligand were responsible for chelation of one mole of uranyl ions. According to kinetic studies, the pseudo–first order model accurately predicted the kinetics of U(VI) extraction by HTIP ligand with a retention power of 245.47 mg/g. The thermodynamic parameters Δ*S*°, Δ*H*°, and Δ*G*° were also calculated; the extraction process was predicted as an exothermic, spontaneous, and advantageous extraction at low temperatures. As the temperature increased, the value of ∆*G*° increased. The elution of uranium ions from the loaded HTIP/CHCl_3_ was achieved using 2.0 mol of H_2_SO_4_ with a 99.0% efficiency rate. Finally, the extended variables were used to obtain a uranium concentrate (Na_2_U_2_O_7_, Y.C) with a uranium grade of 69.93% and purity of 93.24%.

## 1. Introduction

**Scheme 1:**
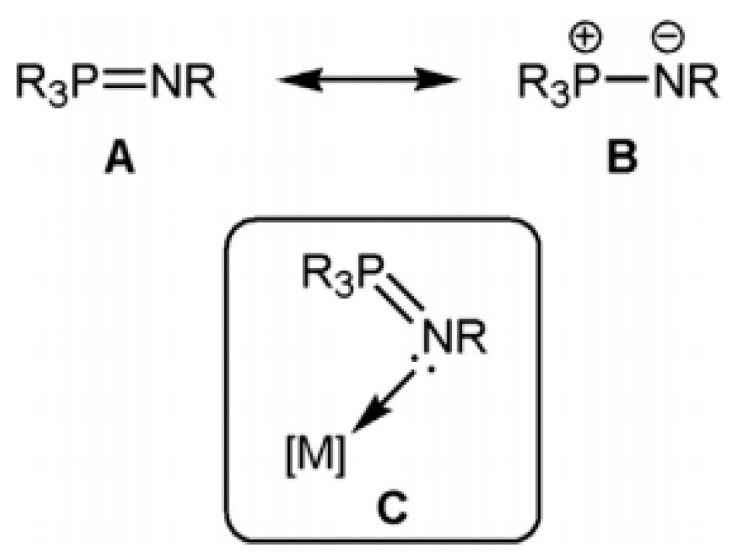
The recognized structures of an iminophosphorane and its coordination to a metal.

The arrangement of the reactive center in a ligand has been the basis for many advances in inorganic as well as organometallic chemistry. Accordingly, significant time and effort have gone into designing and synthesizing ancillary ligands, besides studying the properties, reactivities, and geometries of the resulting complexes. In some cases, these efforts have resulted in new applications in stoichiometric chemistry, catalysis, and materials science [1,2,3]. A large number of structural studies for systems with phosphinimide ligands have been published in this area. These chelating and anionic ligands (R_3_PN) are easily obtained from neutral phosphinimine precursors, prepared utilizing the simple and long–established Staudinger reaction [4,5,6]. Iminophosphorane compounds, also known as phosphoranimines, phosphinimines, or phosphazenes, were detected in 1919; they are organic composites with the general structure R_3_P=NR. Iminophosphoranes contain nitrogen alongside phosphorus atoms, which coordinate to transition metals using the nitrogen atom’s lone pair of electrons. Multidentate ligands are produced by adding extra donor sites into the iminophosphorane ligand, and they are gaining popularity in both coordination chemistry and catalysis [7,8,9]. Iminophosphoranes are resonance hybrids of the two recognized forms A and B, and have a highly polarized P=N bond (Scheme 1). They can also integrate transition metals through the sp^2^–hybridized nitrogen atom’s lone pair, resulting in stable complexes (C in Scheme 1) [10]. Iminophosphoranes, which are primarily s–donor ligands with only minimal p–acceptor characteristics, have a limited inherent coordinating capability since they can be easily substituted by other ligands.

**Scheme 2:**
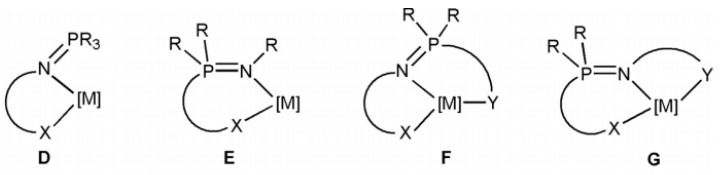
Common structures of complexes of metal with polydentate iminophosphorane–based ligands.

Polydentate mixed ligands, such as those found in the generic metal complex structures shown in Scheme 2 (D–G), can stabilize a wider range of metal ions than their monodentate R_3_P = NR counterparts due to the addition of further donor sites to the iminophosphoranes. The reactivity of the metal center can be modified as needed by selecting and adjusting these new donor sites, as well as the appropriate linkers. It is regarded as a critical component in achieving high levels of activity and selectivity [7].

**Scheme 3:**
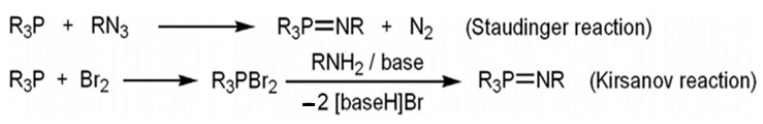
Principal techniques of iminophosphorane synthesis.

Several procedures for the production of iminophosphoranes are recognized; however, the most dominant and extensively used are the Staudinger as well as Kirsanov reaction (Scheme 3). A phosphine (PR_3_) is used as a starting material in both of them. The Staudinger reaction involves direct oxidation with an organic azide, whereas the Kirsanov reaction involves initial bromination and consecutive reaction of the resulting phosphine dibromide compound with a primary amine in the presence of a base [11,12,13].

There are various methods for the synthesis of phosphazenes, such as the free radical process, thermal ring opening polymerization, substitution polymerization and cationic or anionic living polymerization, and solution polymerization method [14,15,16,17]. Phosphazenes have highly tunable chemical as well as physical properties that count on the substituents attached to the phosphorus atom. Therefore, they are employed in an enormous range of fields, such as rechargeable batteries [18], liquid crystals [19], membranes [20] and lubricants [21], anticancer agents [22], flame retardants [23], antibacterial reagents [24], biological materials [25], synthetic bones [26], photosensitive substrates [27], thermally stable macromolecules [28], a high limiting oxygen index (LOI) [29], coordination metal supports and reagents for organometallic chemistry [30], silicon-containing compounds [31], and catalysts [32].

Uranium is a chemical element with the symbol U, which belongs to the actinides. Due to its strategic significance in the energy sector, uranium became the most valuable heavy metal [32,33,34,35]. In nature, the Earth’s crust contains an average of 4.0 mg/L uranium [36]; however, it deposits in different types of rocks. It is a hexavalent state in the secondary minerals, such as kasolite, uranophane, autunite, etc., while it has a tetravalent oxidation state in the form of primary minerals, e.g., coffinite, pitchblende, uraninite, etc. Firstly, uranium is leached from its ore in an acidic or alkaline process, and extracted from the resulting leach liquor using an ion exchanger or a solvent extraction technique [37,38,39,40,41,42,43,44,45,46,47]. Solvent extraction (SX) is a widely used technique for the recovery and separation of base metals and strategic metals in hydrometallurgy. Solvent extraction efficacy is subject to several factors including the type of separation apparatus (pulsed columns, mixer–settler, etc.), the type of feed solution, the applied flowsheets, the chemical composition of the organic solvent, the flow rates of both aqueous and organic phases in the extractions along with elution steps, etc. In the SX method, the extractant plays an essential role. It should have tremendous solubility in the organic solvent. Nevertheless, it must have an extremely low aqueous phase solubility. The solvent ought to be nonvolatile, industrially serviceable, nontoxic, nonflammable, and affordable for the element extraction process [48,49]. Previous studies have used several extractants for uranium extraction by solvent extraction. The derivatives of organophosphorus compounds have been employed since 1900 in uranium extraction processes. Compounds such as Cyanex 302, Cyanex 272, TBP, DEHPA, D2EHPA/TOPO mixtures, Primene JM–T/Alamine–336 mixture [50], DDPA, HDPA, CMPO, DNPPA, and DBBP, which are used to extract uranium from different matrices, were studied [51,52,53,54,55,56]. Several ligands with high selectivity are employed for uptake of uranium ions, comprising organic crown ethers [57], calixarenes [58], and Schiff bases. The long-chain amines have proven to be outstanding extractants for a large number of anionic metal complexes applied widely in uranium removal, such as Adogen–383, TOA, and Aliquate–336 [59,60,61].

Amides are deemed one of the outstanding as well as vital organic functional groups in pharmaceuticals, agrochemicals, polymers, and naturally occurring molecules. In addition, carboxamides have considerable value in coordination, medicinal, and organic chemistry. They can be obtained via the amidation process where a condensation reaction occurs between carboxylic acid and amine. One-pot synthesis of the chelating carboxamides using different catalysts were proposed. For instance, pyridine–2,6–dicarboxylic acid bis–(3–hydroxy phenyl) amide (Pydca) along with tetra–kis (2–ethylhexyl) pyridine–2,6–dicarboxamide (EHPyCA) were successfully synthesized and utilized for removal of thorium as well as uranium from leach liquors of ore samples in Egypt [62,63,64].

In this study, a new synthetic N–hydroxy–N–trioctyl iminophosphorane (HTIP) chelating ligand was synthesized using an effective alternative technique compared to the traditional Staudinger and Kirsanov methods and employed for uranium extraction from acidic solution. Both the removal and elution factors were optimized. Furthermore, the study dealt with the equilibrium, kinetic, and thermodynamic characteristics of uranium extraction from G. Gattar leach fluid, North Eastern Desert of Egypt.

## 2. Materials and Methods

### 2.1. Apparatus

The acidity and alkalinity of solutions were detected using a digital pH meter (VSTAR10 series, Thermo Scientific™, Waltham, MA, USA) with an error of ±0.1. An analytical balance (AUW220D Series, Shimadzu, Kyoto, Japan) with standard deviation of 0.05 mg was utilized to measure all samples. A Vibromatic-384 shaker was employed to mix the contents in separating funnels. The crystal structure of materials was examined using X-ray diffraction (XRD) technique (D8 Discover Family, Bruker, Billerica, MA, USA). Quantitative analysis of U(VI) was executed with a double beam spectrometer (T80 UV/Vis, PG Instrument, Leicestershire, UK) using the arsenazo (III) indicator and 650 nm wavelength against an appropriate standard solution [65]. Furthermore, oxidometric titration against ammonium metavanadate and sodium diphenyl amine sulfonate as an indicator using automatic titrator [66,67] (SCOTT Instrument, GmbH, DE, Sialkot, Pakistan) was also employed to confirm the concentration of U(VI) ions. An ICP-OES spectrometer (OPTIMA 5300 DV, PerkinElmer, Waltham, MA, USA) was applied to specify the concentration of uranium and metal ions of G. Gattar leachate. A Reichert Thermovar was used to determine the melting point. The elemental analysis of uranium concentrate product and HTIP ligand were recorded using EDX (JSM–7900F, Jeol, Tokyo, Japan). An FTIR spectrophotometer (IRPrestige–21, Shimadzu, Kyoto, Japan) was employed to record the IR spectra using KBr disc. The ^1^H and ^13^C–NMR spectra were obtained at 500 MHz using an NMR spectrometer (Bruker Avance TM 500, Bruker, Billerica, MA, USA). The coupling constant (J) was measured in Hertz (Hz), while the chemical shift (δ) was measured in ppm. A mass spectrometer (Finnigan SSQ 7000 spectrometer, Thermo Finnigan, San Jose, CA, USA) was used for the molecular formula. The laboratories of National Research Center (NRC), Cairo, Egypt performed the FTIR, GC–MS, 1H, and 13C-NMR analyses.

### 2.2. Reagents

All of the reagents were made with analytical grade chemicals. HCl, H_2_SO_4_, NaOH, and HNO_3_ were purchased from POCH S.A., Gliwice, Poland. Trioctylphosphine oxide (TOPO) and N–hydroxylamine hydrochloride were obtained from Thermo Fisher Scientific–Acros Organics Inc., Geel, Belgium. Ammonium metavanadate, sodium nitrite, FeSO_4_.7H_2_O, AlCl_3_, and urea were supplied by Scharlau Chemie. S.A., Barcelona, Spain. Uranyl acetate dihydrate, arsenazo III, and sodium diphenyl amine sulfonate were obtained from Merck, Darmstadt, Germany. Furthermore, methanol, DMF, and ethyl acetate were purchased from Fluka, Gillingham, UK. All reactions were performed utilizing flame-dried glassware. Thin paper chromatography (PC) was used to observe the reaction’s development. Ethanol plus ethyl acetate (50:50 *v*/*v*) was adopted as an eluent. A UV lamp was used to visualize spots on the PC plates (250 nm).

### 2.3. Synthesis of N–Hydroxy–N–Trioctyl Iminophosphorane (HTIP) Chelating Ligand

Two primary procedures were used to produce N–Hydroxy–N–trioctyl iminophosphorane (HTIP). The first stage in neutralization was to mix 0.1 mole of NaOH (5.0 g, over the stoichiometric quantity) with 0.1 mole of NH_2_OH.HCl (7.0 g) in 50.0 mL of DMF as diluent. For 2.0 h, the mixture was refluxed at 50 °C. The vital goal of the neutralization phase was to make NH_2_OH more nucleophilic. The second swelling process started with 0.1 mole trioctylphosphine oxide (TOPO, 38.6 g) and 0.1 mole (13.3 g) AlCl_3_ hard Lewis acid in 50.0 mL DMF in a condenser for 2.0 h at 50 °C. Finally, the two additions were added to each other and allowed to condense for 6.0 h at 100 °C. The reaction was monitored using paper chromatography (PC) sheets and a solvent mixture of ethanol plus ethyl acetate 50:50 *v*/*v*. A UV lamp was used to detect the spots. The resulting HTIP appeared as a crystalline white to pale yellow solid with a density of ≈0.943 g/cm^3^. After completion of the reaction, the product was obtained by washing it several times with deionized water to remove any leftover DMF and AlCl_3_. The residue was washed, and the recrystallization procedure was performed with an ethanol/DMF mixture.

### 2.4. Preparation of U(VI) Standard Stock Solution

A 1000 mg/L (4.2 × 10^−3^ mol/L) U(VI) standard stock solution was prepared by dissolving 1.872 g of UO_2_(CH_3_COOH)_2_.2H_2_O in deionized water that had been acidified with 5.0 mL concentrated HNO_3_ to avoid hydrolysis in a 1000 mL volumetric flask. In addition, numerous standard stock solutions of 1000 mg/L of probable different ions during U(VI) extraction by HTIP/CHCl_3_ chelating ligand were generated by dissolving proper amounts of their salts in 1000 mL deionized water.

### 2.5. Extraction and Stripping Procedures

The pH value, shaking time, initial uranium(VI) conc., HTIP conc., temperature, and different ions were all tuned to optimize U(VI) ion extraction from synthetic solution by HTIP/CHCl_3_. In these experiments, 25.0 mL of a 100 mg/L (4.2 × 10^−4^ mol/L) synthetic U(VI) ions solution was mechanically shaken for a predefined period of time with 25.0 mL of varied concentrations of HTIP/CHCl_3_. Both the extraction distribution ratio *D* and stripping ratio *D*′ were calculated using the following Equation (1) [68]:

(1) $$D=\frac{C_{o}}{C_{a}};D^{\prime }=\frac{C_{a}}{C_{o}}$$

where *C_o_* and *C_a_* (mg/L) correspond to the concentration of U(VI) in organic and aqueous phase, respectively. Moreover, the distribution coefficient (*K_d_*) and extraction percentage (*E*%) were calculated using Equations (2) and (3), respectively:

(2) $$K_{d}=\frac{C_{i}-C_{e}}{C_{e}}\times \frac{V_{a}}{V_{o}}$$

(3) $$E\%=\frac{100D}{D+\left(\frac{V_{a}}{V_{o}}\right)}$$

where *C_i_* and *C_e_* (mg/L) symbolise for the initial and equilibrium concentration of U(VI) ions, respectively. *V_o_* and *V_a_* (mL) represent organic and aqueous phase volumes, respectively. Nonetheless, the stripping procedures were performed by shaking different volumes of the loaded organic solvent with the eluent (2.0 mol H_2_SO_4_) for 10 min at ambient temperature. After equilibration, the two layers were entirely separated, and the U(VI) ion concentration was measured. The stripping percentage (*S*%) can be expressed using the next Equation (4):

(4) $$S\%=\frac{100D^{\prime }}{D^{\prime }+\left(\frac{V_{o}}{V_{a}}\right)}$$

### 2.6. Production of G. Gattar Granite Leach Solution

Uranium–rich ore sample was collected from G. Gattar granite, North Eastern Desert, Egypt. Percolation leaching technique was applied; H_2_SO_4_ was used as a leachant. The leaching parameters were optimized at 75.0 g/L of H_2_SO_4_, −100 mesh particle size for 4.0 h leaching time, and 1:1 S/L phase ratio at room temperature. ICP-OES and colorimetric analysis were used to detect the chemical composition of the G. Gattar ore sample and its leachate.

## 3. Results and Discussion

### 3.1. Characterization of N–Hydroxy–N–Trioctyl Iminophosphorane (HTIP) Chelating Ligand

**Scheme 4:**
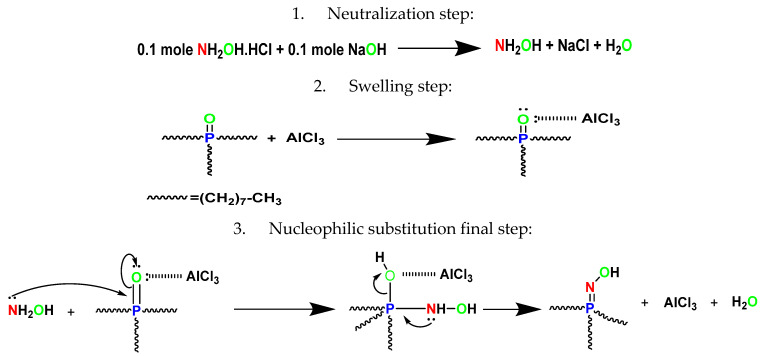
Synthesis of N–Hydroxy–N–trioctyl iminophosphorane (HTIP).

The synthesis procedures for N–Hydroxy–N–trioctyl iminophosphorane (HTIP) chelating ligand and the suggested mechanism of the reaction are illustrated in Scheme 4. A very important clarification should be mentioned concerning the role of the Lewis acid (AlCl_3_) in the fabrication of HTIP ligand. The hard Lewis acid was characterized by a great charge density with vacant orbitals, which could attract electrons from the oxygen phosphine group. This operation facilitated the breaking of –P=O bonds. After that, the nucleophilic attack of NH_2_OH upon the phosphine group could take place easily, as the phosphorous atom acted as an electrophile. This method is considered as an effective alternative technique to the Staudinger and Kirsanov methods.

**Figure 1:**
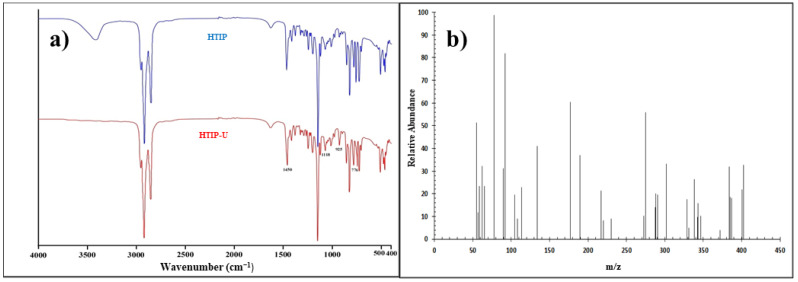
(**a**) FTIR spectra of HTIP and HTIP–U(VI) complex; (**b**) GS–MS spectrum of HTIP chelating ligand.

The obtained yield was ≈40.0 g (≈71.0%) with a melting point equal to 135–140 °C. Numerous functional groups in the synthesized HTIP were predicted using important observations in Fourier transformation infrared spectroscopy (FTIR) [69]. Figure 1a displays the FTIR spectra of the HTIP and its complex with U(VI) ions. The HTIP had a strong peak, centered at roughly 3409.48 cm^−1^, which was linked to the OH group’s stretching mode. After chelation with U(VI) ions, the stretching vibration related to the OH group disappeared, indicating that the OH group took part in the chelation. The features located at 2850–2918, 701.38, 819.83, 1145.18 and 1464.72 cm^−1^ were more likely caused by the CH aliphatic, (CH_2_)_n_ aliphatic, –P=N, –P–N, and N–O bonds, respectively. The frequency of –P=N and –P–N stretching vibrations in the HTIP–U(VI) complex were shifted to a lower frequency as compared with the free ligand (776.3 and 1118.41 cm^−1^), indicating that there was an appreciable chelation between HTIP and U(VI). The peak in the HTIP–U(VI) complex spectrum observed at 925 cm^−1^ corresponded to coordinated U=O bond [40,70].

Gas chromatography–mass spectrometry (GC–MS) is a powerful and effective tool for predicting chemical formulas and purity; the more stable fragment, [m/z]^+^, is an influential and strong tool. The synthetic ligand’s molecular weight was represented by the molecular ion peak with a value of 401.63. Some important fragmentation patterns related to the synthesized HTIP were observed, including [P=N–OH]˙ with a molecular weight of 61.98, [P=N–O]˙ with a molecular weight of 60.98, [CH_3_(CH_2_)_7_]˙ with a molecular weight of 113.25, and a fragment with a 384.66 molecular weight, which denoted the formation of the [(CH_3_(CH_2_)_7_)_3_P=N]˙ moiety. The results of the entire investigation indicated that the HTIP ligand could be synthesized successfully. Figure 1b demonstrates the GC–MS spectrum of the HTIP chelating ligand.

**Figure 2:**
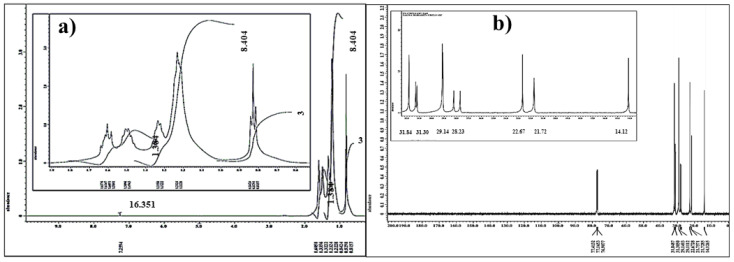
(**a**) ^1^H–NMR spectrum; (**b**) ^13^C–NMR spectrum of HTIP chelating ligand.

^1^H–NMR analysis with a 500.15 MH_Z_ energy and CDCl_3_ as a diluent is a useful and efficient technology that provides important information about protons in the produced substance and aids in structure estimates. The primary δ (ppm) assignments were 7.259, 0.815–0.842, and 1.222–1.637 ppm, which corresponded to the protons of OH, methyl, and methylene, respectively. The assignments of the OH proton (δ = 7.259 ppm) were more deshielded than the assignments of the –CH_2_ and –CH_3_ protons, whereas –CH_2_ (δ = 1.222–1.637 ppm) was more deshielded than –CH_3_ (δ = 0.815–0.842 ppm). Figure 2a illustrates the ^1^H–NMR characterization of the HTIP chelating ligand.

^13^C–NMR analysis with a 125.76 MH_Z_ energy and CDCl_3_ as a diluent is a useful method for determining the number of carbon atoms in an HTIP ligand. The major δ (ppm), which is connected to alkyl carbon, occurred at 14.126–31.848 ppm (Figure 2b). The –CH_3_ carbon appeared at 14.126 ppm, which was more protected than the other –CH_2_ carbons that appeared in chemical shift ranges of 21.728–31.848 ppm.

**Figure 3:**
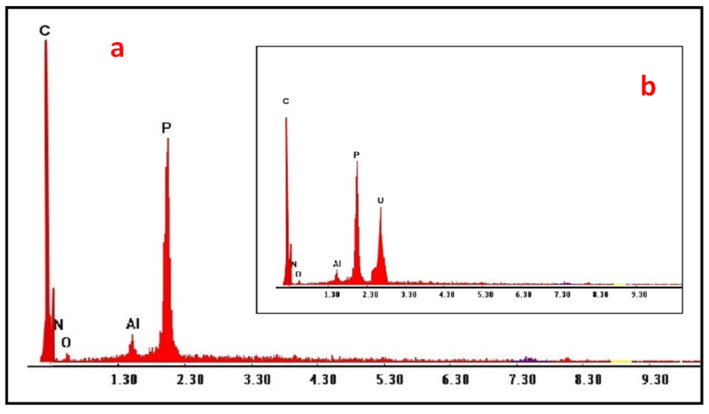
EDX spectra of (**a**) HTIP; (**b**) HTIP–U(VI) complex.

A characteristic EDX analysis was performed to illustrate the elements composing HTIP chelating ligand after and before uranyl ion chelation. A significant peak from 0–0.5 keV represented carbon, oxygen and nitrogen atoms, while the peak at 1.65 keV represented the phosphorous atom. The appearance of uranium gave an obvious sign for the chelation of uranyl ions by HTIP chelating ligand. The identification of HTIP and HTIP–U(VI) complex by EDX spectrum is presented in Figure 3.

### 3.2. Extraction Procedures

#### 3.2.1. The Influence of pH

**Figure 4:**
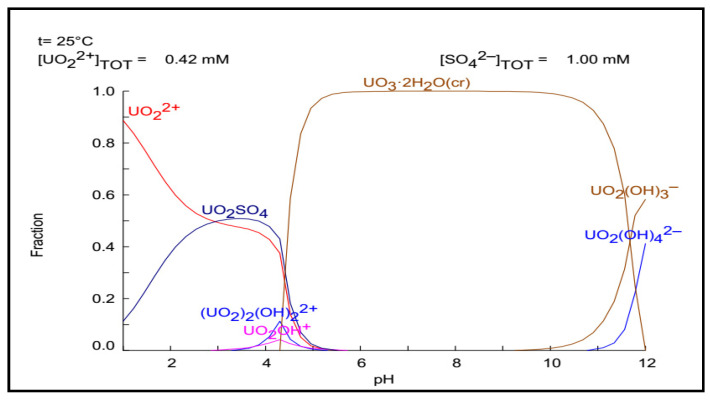
The HYDRA–MEDUSA diagram of uranyl ions at different pH values.

The role of pH was very important in the retention of U(VI) on the ligand’s active sites because it impacted the form of uranium in aqueous media as well as the features of the active sites of the HTIP ligand. Figure 4 shows the species of uranyl ions in the HYDRA–MEDUSA program at different pH vales. There are many U(VI) species in solution, according to earlier research [71,72]. Uranium can be found in cationic, neutral, or anionic species. Until pH 5.0, uranium is primarily present in the cationic forms UO_2_^2+^, UO_2_(OH)^+^, and (UO_2_)_2_(OH)_2_^2+^, whilst UO_2_SO_4_, UO_3_.2H_2_O, UO_2_(OH)^3–^, and UO_2_(OH)_4_^2–^ are neutral and anionic species that are identified till pH 12.0.

**Figure 5:**
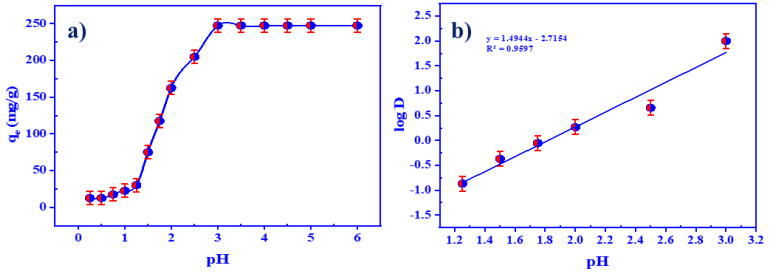
(**a**) The influence of pH on the retention of uranium by HTIP chelating ligand; (**b**) the regression analysis of U(VI) ion extraction by HTIP at various pH values. (U(VI) conc.: 0.42 × 10^−4^ mol/L, HTIP/CHCl_3_ conc.: 0.99 × 10^−3^ mol/L (10 mg/25 mL), A/O 1:1, T: 25 °C, time: 30 min).

The retention of U(VI) on HTIP was examined using 25.0 mL of a 4.2 × 10^−4^ mol/L U(VI) solution (100 mg/L) and 25.0 mL of 0.99 × 10^−4^ mol/L HTIP/CHCl_3_ (10 mg of HTIP in 25.0 mL CHCl_3_) at pH ranges from 0.25–6.0 and room temperature for 30 min. Figure 5a depicts the acquired data, which reveal that the retention capacity was enhanced from pH 0.25 (*q_e_* = 12.5 mg/g) to pH 3.0 (*q_e_* = 247.5 mg/g) and remained constant up to pH 6.0. It was noticed that in highly acidic medium, pH 0.25–1.0, there was a small variation in HTIP retention due to the high conc. of hydrogen ions, which may compete with uranyl ions and protonated HTIP ligand. The ultimate retention of U(VI) was detected at pH 3.0 (*q_e_* = 247.5 mg/g) because UO_2_^2+^, UO_2_(OH)^+^, and (UO_2_)_2_(OH)_2_^2+^ species are predominant in this range. Therefore, pH 3.0 has been proposed as the best pH value for U(VI) ion retention on HTIP/CHCl_3_, with *q_e_* = 247.5 mg/g retention capacity (99.0%). A graph of log*D* versus pH shows a straight line with a slope of 1.5 and an intersection of 2.7157 in linear regression analysis (slope analysis), as displayed in Figure 5b. The value of slope represents the amount of hydrogen ions set free in the aqueous medium during the formation of the HTIP–U(VI) complex, indicating that about 1.5 moles of hydrogen ions were released during the extraction procedure. Moreover, at pH value 3.0 (logβ = 2.7157), the stability constant (β) of the HTIP–U(VI) complex was computed and found to be 519.63; it showed that the HTIP ligand had a high affinity for uranyl ions.

**Scheme 5:**
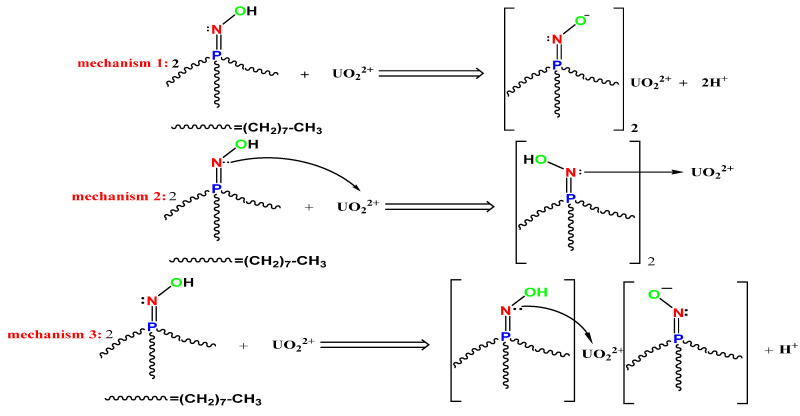
Proposed mechanisms of UO_2_^2+^ ion extraction by HTIP chelating ligand.

Lastly, based on the preceding analysis, the suggested complex structure and chelation mechanism are presented in Scheme 5. It is clear that the first mechanism mainly depends on the pH value. Competition occurs in highly acidic media between both hydrogen and uranyl ions, causing the tendency of equilibrium to shift towards the left, but in slightly acidic, neutral and alkaline medium, may cause hydrogen ion withdrawal with increased chelation effect and uptake capacity of the HTIP ligand. The second mechanism depends on pH value and the affinity of the HTIP ligand for uranyl ions. The third mechanism is a mixture between the two later mechanisms.

#### 3.2.2. The Influence of Equilibration Time

**Figure 6:**
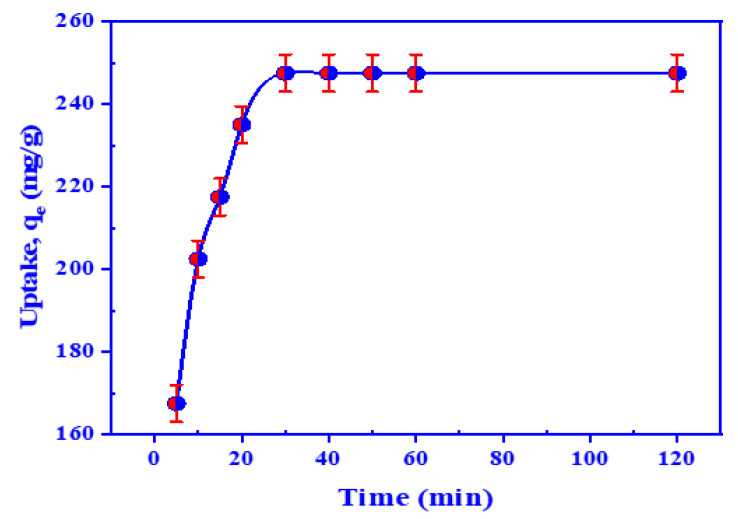
The influence of time on the retention of uranium by HTIP chelating ligand. (U(VI) conc.: 0.42 × 10^−4^ mol/L, HTIP/CHCl_3_ conc.: 0.99 × 10^−3^ mol/L (10 mg/25 mL), pH 3.0, A/O 1:1, T: 25 °C).

Contact time is one of the highly essential factors on the financial side. The effect of equilibrium time on U(VI) retention was examined using 9.96 × 10^−4^ mol/L (10 mg/25.0 mL) HTIP/CHCl_3_ and a 25.0 mL aqueous uranium(VI) ion solution with a concentration of 0.42 ×10^−3^ mol/L at pH 3.0. U(VI) ion retention rose with rising equilibrium time and reached an ultimate value (247.5 mg/g, 99.0%) after 30 min, which remained roughly constant for the next 120 min, as shown in Figure 6. As a result, 30 min was deemed sufficient for achieving equilibrium in subsequent testing, and it was applied in all successive investigations.

**Figure 7:**
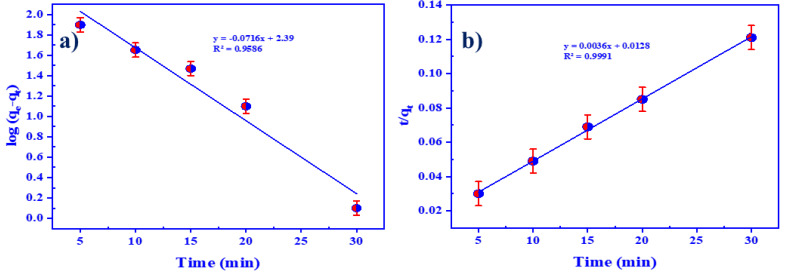
(**a**) PFO Kinetic model; (**b**) PSO kinetic model of U(VI) ion extraction by HTIP chelating ligand. (U(VI) conc.: 0.42 × 10^−4^ mol/L, HTIP/CHCl_3_ conc.: 0.99 × 10^−3^ mol/L (10 mg/25 mL), pH 3.0, A/O 1:1, T: 25 °C).

Kinetic studies were used to express the rate of uranyl ion entrapment using HTIP/CHCl_3_, providing crucial information for the design and modeling of the extraction process. Both pseudo first order (PFO) and second order (PSO) kinetic models were employed to identify the mechanism of U(VI) ion entrapment using HTIP/CHCl_3_ and the rate constant of the process. The PFO model is presented by the equation below [73]:

(5) $$\log\left(q_{e}-q_{t}\right)=\log q_{e}-\left(\frac{k_{1}t}{2.303}\right)$$

where *q_e_* and *q_t_* (mg/g) correspond to the quantity of U(VI) ions entrapped per unit mass at equilibrium and time *t*, respectively, and *k*_1_ (min^−1^) denotes the rate constant. Figure 7a displays a straight line plot of log(*q_e_ − q_t_*) against *t*; the values of both *k*_1_ and *q_e_* were calculated from the slope and intercept, respectively. The calculated value of *q_e_* was 245.47 mg/g, and of *k*_1_, 0.1648 min^−1^, with *R*^2^ = 0.9586. It is obvious that the calculated value of *q_e_* was extremely close to the practical retention capacity of 247.5 mg/g (Table 1). Nonetheless, the PSO kinetic model was calculated using the equation below [74]:

(6) $$\frac{t}{q_{t}}=\frac{1}{k_{2}q_{e}^{2}}+\frac{t}{q_{e}}$$

where *k*_2_ (g/mg.min) is in agreement with the rate constant. Figure 7b demonstrates the straight line of graphing *t/q_t_* vs. *t*; it has a slope of 1/*q_e_* and an intercept of 1/*k*_2_*qe*^2^. The PSO model was found to be ineffective in explaining the practical data. Table 1 illustrates the calculated value of *q_e_* as 277.47 mg/g, which was somewhat greater than the experimental retention capacity, whereas the value of *k*_2_ was 0.001 min^−1^ with *R*^2^ equal to 0.9991. Therefore, the PFO kinetic model was more reliable in characterizing the extraction process of U(VI) ions using HTIP/CHCl_3_, as it was appropriate for the experimental data.

#### 3.2.3. The Impact of Initial U(VI) Ion Concentration

**Figure 8:**
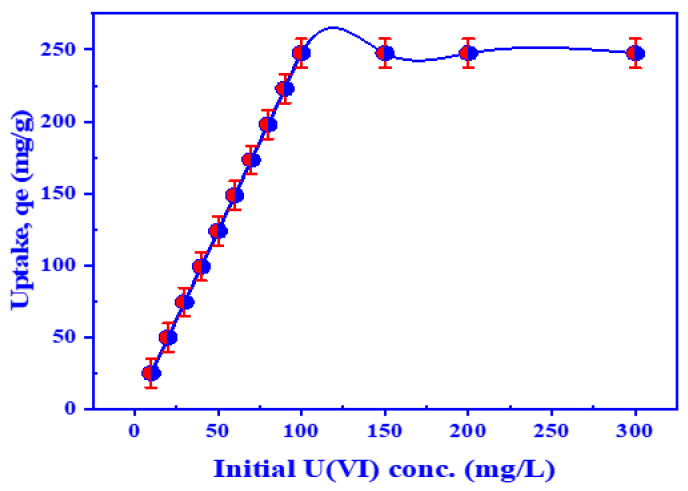
The influence of initial U(VI) ion concentration on the retention of uranium by HTIP chelating ligand. (pH 3.0, HTIP/CHCl_3_ conc.: 0.99 × 10^−3^ mol/L (10 mg/25 mL), time: 30 min, A/O 1:1, T: 25 °C).

The impact of the initial concentration of U(VI) ions on extraction efficiency is crucial to investigate since it helps us to anticipate retention power. A diagram of retention power against different initial U(VI) concentrations is shown in Figure 8. Two different stages can be noted. In the first stage, the retention power of HTIP ligand increases conspicuously from 24.75 to 247.5 mg/g with the rising initial concentration of U(VI) ions because the number of active points on the HTIP ligand exceeds the number of uranyl ions in solution. On the other hand, the retention capacity remains constant in the second stage, when the concentration of uranyl ions increases from 100 to 300 mg/L U(VI), because uranyl ions have entirely interacted with the HTIP active sites. The number of active HTIP sites is less than that of uranyl ions. At an initial U(VI) concentration of 100 mg/L, the maximum value of U(VI) ion retention on HTIP/CHCl_3_ is 247.5 mg/g (99.0%).

#### 3.2.4. The Influence HTIP Dose

**Figure 9:**
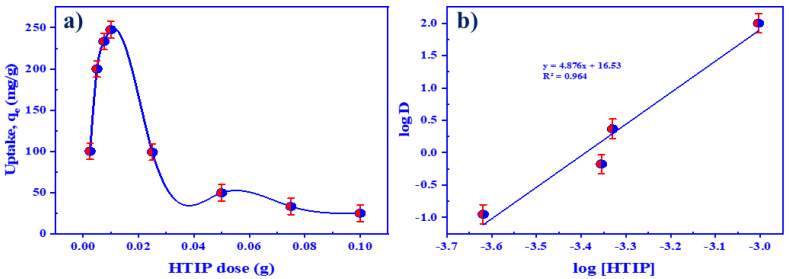
(**a**) The influence of HTIP dose on the retention of uranium; (**b**) the regression analysis of U(VI) ion extraction by HTIP at different concentrations. (U(VI) conc.: 0.42 × 10^−4^ mol/L, pH 3.0, Time: 30 min, A/O 1:1, T: 25 °C).

The HTIP amount is important for better uranyl ion sequestration because it impacts the equilibrium of the system. The effect of HTIP doses varying from 0.0025 to 0.1 g/25.0 mL CHCl_3_ on U(VI) retention efficiency was investigated. Figure 9a shows that augmenting the HTIP quantity from 0.0025 to 0.01 g/25.0 mL CHCl_3_ improved U(VI) ion retention efficiency, followed by retention diminishing from 0.025 g to 0.1 g/25.0 mL CHCl_3_ because the number of HTIP incorporation sites surpassed the number of uranyl ions. The retention capacity of 0.01 g/25.0 mL HTIP/CHCl_3_ was 247.5 mg/g with a 99.0% removal efficiency. As a consequence, the best concentration for consequent extraction studies was 0.01 g/25.0 mL HTIP/CHCl_3_. It is obvious that the retention efficiency of HTIP is higher than that of other materials reported in earlier studies, as shown in Table 2.

The regression analysis was studied to guarantee the configuration of the HTIP–U(VI) complex formed. Figure 9b illustrates a straight line with a slope of 4.876 and *R*^2^ = 0.964 when plotting log*D* versus logHTIP. The linear regression analysis (slope analysis) can elaborate the stoichiometry mechanism between HTIP chelating ligand and U(VI) ions, which suggests that 1.0 mole of U(VI) ions is chelated by 4.0 moles of HTIP.

#### 3.2.5. U(VI) Ion Distribution Isotherm (McCabe–Thiele Isotherm)

**Figure 10:**
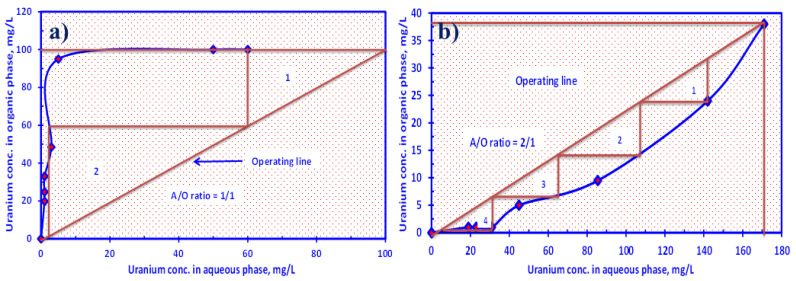
McCabe–Thiele diagram of (**a**) U(VI) ion extraction by HTIP chelating ligand; (**b**) U(VI) ion stripping isotherm from HTIP–U(VI) complex. (U(VI) conc.: 0.42 × 10^−4^ mol/L, HTIP/CHCl_3_ conc.: 0.99 × 10^−3^ mol/L (10 mg/25 mL), pH 3.0, time: 30 min, T: 25 °C).

Figure 10a demonstrates the McCabe–Thiele diagram of the extraction distribution isotherm of U(VI) ions by HTIP in a system consisting of U(VI) concentration 0.42 × 10^−4^ mol/L, HTIP/CHCl_3_ conc. 0.99 × 10^−3^ mol/L (10 mg/25.0 mL), 3.0 pH value, and A/O phase ratio 1:1 for 30 min. It was observed that two theoretical extraction phases are required to extract nearly all of the U(VI) ions. Furthermore, the elution of U(VI) ions from the HTIP–U(VI) complex was conducted using 2.0 M of H_2_SO_4_. The HTIP/CHCl_3_ organic phase had a concentration of U(VI) ions of 95.0 mg/L. Figure 10b depicts the McCabe–Thiele diagram of the U(VI) ion stripping distribution isotherm; to release almost all of the entrapped U(VI) ions from the HTIP–U(VI) complex, four theoretical stripping stages are required with a 2:1 A/O phase ratio.

#### 3.2.6. The Influence Temperature

**Figure 11:**
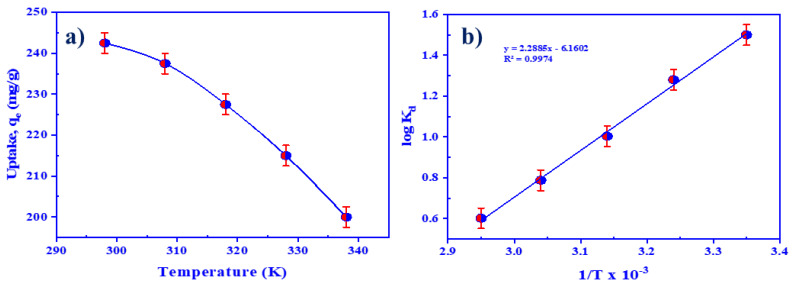
(**a**) The influence of temperature on the retention of uranium by HTIP chelating ligand; (**b**) thermodynamic study of U(VI) ion extraction by HTIP chelating ligand (U(VI) conc.: 0.42 × 10^−4^ mol/L, HTIP/CHCl_3_ conc.: 0.99 × 10^−3^ mol/L (10 mg/25 mL), pH 3.0, A/O 1:1, time: 30 min).

The effectiveness of temperature on the removal of U(VI) ions using HTIP/CHCl_3_ was investigated by mixing 25.0 mL of 0.99 × 10^−4^ mol/L (10 mg/25.0 mL) HTIP/CHCl_3_ and 25.0 mL of uranium(VI) ion solution with a concentration of 4.2 × 10^−4^ mol/L (100 mg/L) for 30 min at pH 3.0 and agitation temperature varying from 25–65 °C. The uptake efficiency of HTIP/CHCl_3_ for U(VI) ions dropped from 247.5 mg/g to 200 mg/g when the temperature was elevated from 25 to 65 °C, (Figure 11a). The pattern suggests that the process of extracting U(VI) ions through HTIP is exothermic.

Thermodynamic analysis was also carried out to characterize the extraction mechanism using the Gibbs free energy formula [82,83,84]:

(7) ∆G°=∆H°−T∆S°

(8) $$\log K_{d}=\frac{∆S{}^{\circ}}{2.303R}-\frac{∆H{}^{\circ}}{2.303RT}$$

where ∆*G*° (kJ/mol) points to Gibbs free energy, ∆*H*° (kJ/mol) is attributed to enthalpy change, ∆*S*° (J/mol.K) is defined as entropy change, *T* (K) stands for the temperature, and *R* (8.314 J/mol.K) corresponds to the universal gas constant. Figure 11b displays a log*K_d_* versus 1/*T* graph with *R*^2^ = 0.9974; ∆*H*° and ∆*S*° were generated by the slope and intercept, respectively.

The fact that ∆*G*° is negative implies that the retention of U(VI) on HTIP is thermodynamically spontaneous and attainable (Table 3). In addition, the rise in ∆*G*° values as temperature rises, from −8.646 kJ/mol at 298 K to −3.926 kJ/mol at 338 K, suggests that the retention of uranium at low temperature is desirable. Moreover, the negative value of ∆*H*° indicates that U(VI) ion retention on HTIP is an exothermic process, suggesting that heat is produced during the extraction. Finally, the negative value of ∆*S*° affirms that the capture of U(VI) ions on HTIP is more practicable and less disorganized.

The Arrhenius equation was employed to predict the activation energy (*Ea*) of entrapped U(VI) ions at various temperatures using the slope of the straight line in Figure 11b. The Arrhenius equation was estimated by the subsequent equation [85]:

(9) $$\log K_{d}=\frac{-2.303E_{a}}{RT}+\log A$$

where *E_a_* (kJ/mol) corresponds to the activation energy of extraction and A correlates with the pre-exponential factor, which is independent of temperature. According to calculations, the extraction of U(VI) ions on HTIP ligand requires an activation energy of −8.261 kJ/mol; this implies that the extraction procedure occurs spontaneously and exothermically at room temperature, with no need for activation energy.

#### 3.2.7. The Influence of Co-Ions

The investigated co-ions were identified as co-ions accompanied by U(VI) during the leaching process. The impact of co-existing ions was examined separately under optimal extraction conditions by introducing each one into 25.0 mL of a 4.2 × 10^−4^ mol/L (100 mg/L) U(VI) ion solution. Each ion was identified as an interfering ion when the extraction efficiency differed by more than ±5.0%. Thus, the co-ion concentration that produced a ±5.0% error in uranyl extraction efficiency was used to set the tolerance limit. Table 4 shows that none of the studied co-ions had a negative impact on the retention of U(VI) ions. The findings emphasize the selective extraction of U(VI) on HTIP chelating ligand, which could be used to extract U(VI) ions from leachates of ores in the presence of other ions.

### 3.3. Stripping and Precipitation

Three types of acids with different concentrations ranging from 0.025–2.0 M were utilized as stripping agents for stripping of U(VI) ions from the HTIP–U(VI) complex; the experiments were carried out using 10.0 mL acid volume for 25.0 mL of HTIP–U(VI) complex, shaking well for 10 min at 25 °C. According to the data listed in Table 5, the U(VI) stripping efficiency dropped at low acidic concentrations, but increased when the acid concentration was higher. It was notable that 99.0% stripping efficiency could be achieved with 10.0 mL of 0.5 M HCl, 0.5 M HNO_3_, or 2.0 M H_2_SO_4_. Subsequently, the eluted solution was precipitated with 30.0% NaOH solution until pH 7.0–8.0 was attained, where uranium precipitated as sodium diuranate (Na_2_U_2_O_7_), yielding a final uranium concentrate (yellow cake). The precipitate was left to settle for 24.0 h and filtered. Lastly, the precipitate was dried for 3.0 h at 110 °C in an electrical oven to obtain the uranium concentrate as a final product.

### 3.4. Case Study: U(VI) Recovery from G. Gattar Ore Sample by HTIP Chelating Ligand

The previous data suggest that the HTIP chelating ligand can extract uranium from leachates of geological ores. Accordingly, this hypothesis was investigated using G. Gattar granite leach liquor [85,86]. Uranium (1340 mg/kg) was leached from a G. Gattar ore sample using the percolation leaching technique. The leachate contained approximately 0.45 g/L (450 mg/L) of uranium ions in the presence of a variety of metal ion impurities (Table 6 and Table 7). The recovery experiment was carried out by mixing 1.0 L of HTIP/CHCl_3_ (0.99 × 10^−3^ mol/L) with 1.0 L of leach liquor under the previously defined optimum conditions (pH 3.0, 1:1 A/O phase ratio, 30 min, and 25 °C). According to the study, the extraction efficiency of U(VI) reached 99.0%. In addition, it was affirmed that the released U(VI) ions from the HTIP–U(VI) complex could be easily eluted in 10 min using 2.0 M H_2_SO_4_.

**Figure 12:**
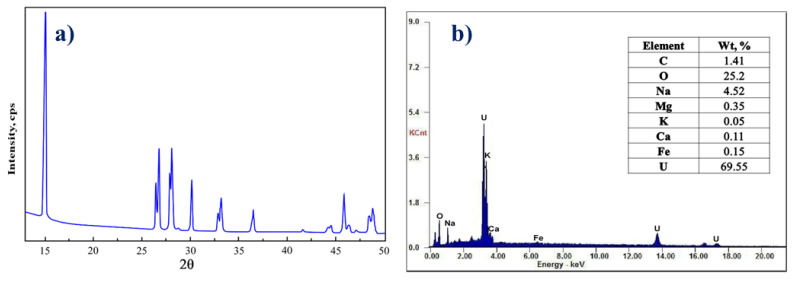
(**a**) XRD spectrum; (**b**) EDX spectrum of Na_2_U_2_O_7_ product from G. Gattar granite ore sample.

**Figure 13:**
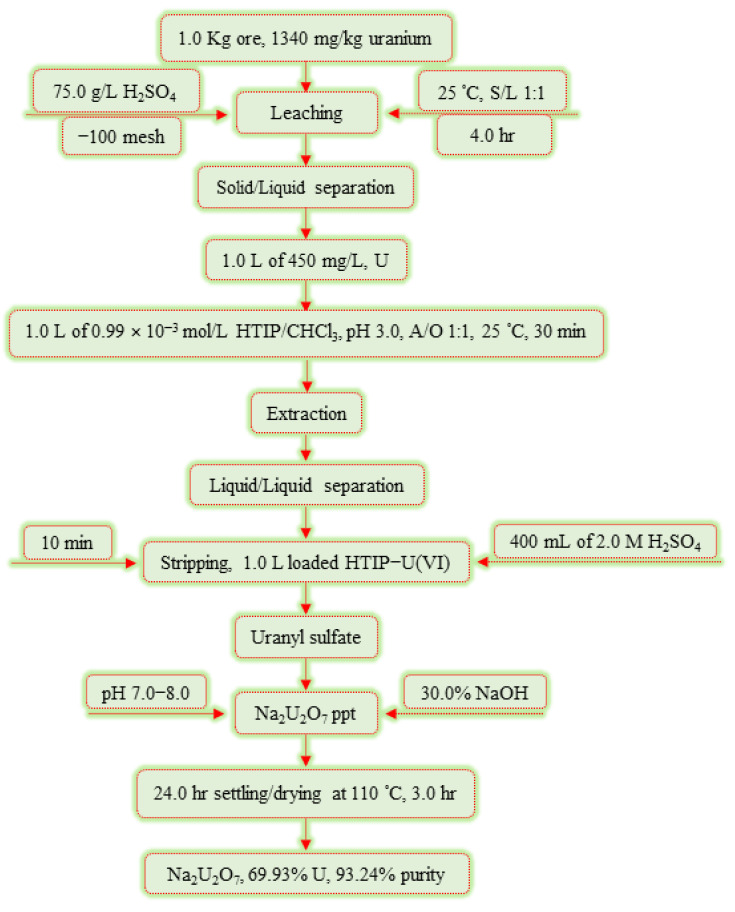
Flow chart illustrating the production of Na_2_U_2_O_7_ from G. Gattar granite ore sample using HTIP chelating ligand.

After the elution process, 30.0% NaOH was used to precipitate U(VI) ions as sodium diuranate precipitate Na_2_U_2_O_7_ by adjusting the pH to 7.0–8.0. The uranium concentrate was characterized using XRD and EDX, in addition to ICP-OES analysis techniques in order to determine the U(VI) content alongside other associated metal ions. The results are given in Figure 12, as well as Table 8. According to the analyses, the uranium(VI) content in the uranium concentrate product “yellow cake’’ was 69.93%, with a purity of 93.24%. Figure 13 demonstrates a flow chart of the recovery of U(VI) ions from G. Gattar granite ore mineralization using HTIP chelating ligand.

## 4. Conclusions

A novel synthetic chelating agent, N–hydroxy–N–trioctyl–iminophosphorane (HTIP), was synthesized using an effective substitutional technique compared to the conventional methods and was utilized to uptake U(VI) ions from leach liquor of G. Gattar, North Eastern Desert, Egypt. Characterization was performed successfully using numerous analytical techniques, including ^1^H–NMR, ^13^C–NMR, FTIR, EDX, and GC–MS analyses. The uranium sequestration procedures were optimized by mixing 25.0 mL of U(VI) ion solution containing 0.42 × 10^−4^ mol/L with 0.99 × 10^−3^ mol/L HTIP/CHCl_3_ at pH 3.0, 1:1 A/O phase ratio, at 25 °C for 30 min. The utmost retention capacity of HTIP/CHCl_3_ was 247.5 mg/g. From the stoichiometric calculations, approximately 1.5 hydrogen atoms were released during the extraction at pH 3.0, and 4.0 moles of HTIP ligand were responsible for chelation of 1 mole of uranyl ions. The kinetic modeling data were well-suited to the pseudo first-order model. Furthermore, thermodynamic study demonstrated a negative Δ*S*° value, indicating that the uptake process is less disordered. Moreover, the rise in Δ*G*° value pointed to the spontaneousness and possibility of removing U(VI) ions at low temperatures. The elution of uranium loaded on HTIP was achieved using 2.0 M of H_2_SO_4_ as uranyl sulfate with 99.0% efficiency. Lastly, uranium concentrate (Na_2_U_2_O_7_, Y.C) with a purity of 93.24% was obtained by adding 30.0% NaOH to the elution and adjusting the pH to 7.0–8.0 with continuous stirring for 2 h.

## Figures and Tables

**Table 1:** The kinetic constants of U(VI) ion extraction by HTIP chelating ligand.

Extraction Capacity *q_e_*, mg/g	PFO			PSO		
	*q_e_*	*k*_1_	*R* ^2^	*q_e_*	*k*_2_	*R* ^2^
247.5 mg/g	245.47	0.1648	0.9586	277.47	0.001	0.9991

**Table 2:** Comparison of retention efficiency of different materials.

Materials	Retention Efficiency, mg.g^−1^	Reference
Poly (aminophosphonic)–functionalized poly (glycidyl methacrylate)–magnetic nanocomposite	262.5	[75]
Nitrogen-enriched carbon–nitrogen polymer, C_3_N_5_	207.0	[76]
Cyanex 923 loaded polymer beads	54.5	[77]
2–Hydroxy–4–aminotriazine–anchored activated carbon	135.0	[78]
D2EHPA–TOPO/SiO_2_–P	48.0	[79]
D2EHPA–TOPO@MCM–41	2.88	[80]
Eggplant (*Solanum melongena*) leaves	110.97	[81]
HTIP	247.5	This study

**Table 3:** Thermodynamic analysis of U(VI) ion extraction by HTIP chelating ligand.

∆*S*°, J/mol.K	∆*H*°, kJ/mol	∆*G*°, kJ/mol				
−118 × 10^−6^	−43.81	298 K	308 K	318 K	328 K	338 K
		−8.645	−7.466	−6.286	−5.106	−3.926

**Table 4:** The influence of co-ions on U(VI) ion extraction by HTIP chelating ligand.

Co-Ions	Tolerance Limit *, mg/L	E%	Co-Ions	Tolerance Limit *, mg/L	E%
Si^4+^	3000	99.0	Ba^2+^	1000	95.0
K^+^	3000	99.0	Ni^2+^	550	95.0
Na^+^	3000	99.0	Rb^+^	650	95.0
Al^3+^	3000	99.0	Pb^2+^	1000	95.0
Ca^2+^	1500	95.0	Mo^6+^	850	95.0
Mg^2+^	1500	95.0	Zr^4+^	950	95.0
Fe^3+^	750	95.0	Sr^2+^	700	95.0
P^5+^	3000	99.0	V^5+^	1000	95.0
Ti^4+^	850	95.0	Zn^2+^	900	95.0

* Tolerance limit: the conc. of co-ion that produces an error does not exceed ± 5.0%.

**Table 5:** Effect of stripping agent conc. on U(VI) ion elution from HTIP–U(VI) complex.

Acid Conc., (M)	Stripping Efficiency, (%)		
	HNO_3_	HCl	H_2_SO_4_
0.025	63.51%	58.6%	57.3%
0.05	85.7%	69.8%	68.2%
0.1	95.8%	93.5%	75.4%
0.5	99.0%	99.0%	89.3%
1.0	99.0%	99.0%	96.2%
2.0	99.0%	99.0%	99.0%

**Table 6:** Chemical composition of G. Gattar granite ore sample.

Major Oxides	Wt., %	Elements	Conc., mg/kg
SiO_2_	48.60	U^6+^	1340
Al_2_O_3_	8.16	Ni^2+^	34
FeO	0.14	Rb^2+^	404
Fe_2_O_3_	2.59	Sr^2+^	38
TiO_2_	0.15	Y^3+^	64
MnO	0.07	Cu^2+^	65
K_2_O	3.38	Zn^2+^	200
CaO	6.73	Ba^2+^	432
Na_2_O	2.35	Zr^4+^	170
P_2_O_5_	0.02	Nb^5+^	25
MgO	0.60	Pb^2+^	36
		Mo^6+^	228

**Table 7:** The chemical analysis of G. Gattar granite leach liquor.

**Metal ions**	Si^4+^	Al^3+^	Fe^3+^	Mo^6+^	V^5+^	P^5+^	Na^+^	K^+^	Zr^4+^	Ti^4+^	U^6+^
**Conc., g/L**	2.33	3.11	1.31	0.06	0.11	0.07	2.11	2.87	0.05	0.03	0.45

**Table 8:** ICP-OES analysis of Na_2_U_2_O_7_ product from G. Gattar granite ore sample.

Elements	Content, (%)	Elements	Content, (%)
U^6+^	69.93	Cr^6+^	0.0031
Na^+^	4.041	Cd^2+^	0.0007
Al^3+^	0.0158	Si^4+^	0.0091
Fe^3+^	0.0207	K^+^	0.0185
Mg^2+^	0.0391	Zr^4+^	0.0035
Ca^2+^	0.0521	Pb^5+^	0.0028
Co^2+^	0.0053	Zn^2+^	0.0037
Cu^2+^	0.0023	V^5+^	0.0035
Ni^2+^	0.0031	Mn^2+^	0.0031

## Data Availability

Not applicable.
